# Strategies for the prevention of periodontal disease and its impact on general health: Latin America and the Caribbean Consensus 2024

**DOI:** 10.1590/1807-3107bor-2024.vol38.0120

**Published:** 2024-11-22

**Authors:** Andres DUQUE DUQUE, Alejandra CHAPARRO PADILLA, Mariana Linhares ALMEIDA, Rubiel Antonio MARÍN JARAMILLO, Hugo Jorge ROMANELLI, Gloria Inés LAFAURIE VILLAMIL

**Affiliations:** (a) CES University, School of Dentistry, Department of Periodontics, Medellín, Colombia.; (b) Universidad de Los Andes, Facutad Filosofia y Humanidades, Department of Dentistry, Santiago, Chile; (c) Faculdade de Enfermagem Nova Esperança, Mossoró, RN, Brazil.; (d) Maimonides University, Faculty of Health Sciences, Department of Periodontics, Buenos Aires, Argentina.; (e) Universidad El Bosque, School of Dentistry, Unit of Basic Oral Investigation, Bogotá, Colombia.

**Keywords:** Periodontal Diseases, Public Health, Latin America, Caribbean Region

## Abstract

Periodontal diseases are closely related to non-communicable diseases, and their prevention depends on their link with healthy lifestyle programs. The purpose of this consensus was to summarize and propose preventive strategies at the community, individual, and research levels in Latin America and the Caribbean. A critical review and search strategy was carried out in Pubmed, LILACS, and SCIELO on three topics: a) Social determinants, risk factors, and behavioral changes related to PD throughout the lives of individuals; b) Impact of mechanical and chemical control of plaque for the prevention of gingivitis; c) Impact on prevention of systemic diseases. Relative to public health policies, no consensus was reported in the region. In some countries, periodontal educational strategies, mainly for pregnant women and for other chronic diseases have been implemented, but their impact on primary and secondary prevention has hardly been evaluated. In recent years, a positive aspect has been the implementation of some public policies, including clinical practice guidelines and care pathways. Based on the latest consensus, multicenter educational research and technological strategies were found in the region, but their effectiveness needs to be evaluated in clinical studies. A barrier to the implementation of preventive strategies has to do with the human factor. Therefore, the training of periodontists to be experts in communication strategies, technologies that allow the empowerment of patients for taking care of their periodontal health are required . Moreover, it is necessary to train professionals from other areas of health, who are more aware of the importance of oral health as a healthy lifestyle.

## Introduction

Periodontal diseases (PD) are inflammatory by nature, and are influenced by host factors, dysbiotic biofilm, in addition to being closely related to non-communicable diseases (NCDs) and other risk factors. By 2030, the FDI’s vision is that oral health data will be integrated into medical data management systems.^1^ Therefore, preventive strategies and recommendations must address the effects of both inflammation, infection, as well as identify predisposing and modifying factors relative to their onset and progression.^2^ In 2020, the Latin American (LATAM) and Caribbean periodontal Consensus, organized by the Latin American Oral Health Association (LAOHA), proposed a regional plan for the prevention of PD at both the individual and community levels. At that time, recommendations for preventing PD included a focus not only on the mechanical and chemical control of the biofilm but also on modifiable risk factors within the causal chain throughout the health-disease process.^3,4^

In PD, primary prevention refers to a preclinical stage to prevent the onset of the disease by reducing the risk factors of biofilm development and gingival inflammation. Secondary prevention concerns properly managing the disease at an early stage, based on timely diagnosis and prompt treatment.^3^ In LATAM and Caribbean countries, where a large part of the population has low and middle income, limited access to health services, and lack of knowledge about diseases and their prevention, the actions taken to reduce the impact of these diseases are especially relevant. In the region, efforts have been made relative to these actions, however, it is necessary to transfer these findings into daily public and private practice and generate new oral health policies focused on preventive strategies. The purpose of this consensus was to propose community, individual, and research strategies for the prevention of PD and its impact on general health, thereby promoting healthy habits from childhood through to the elderly population, based on three topics: a) Social determinants, risk factors, and behavioral changes related to PD throughout the individuals’ life-course; b) Impact of mechanical and chemical control of plaque and bleeding on the prevention of gingivitis; c) Preventive impact of controlling systemic diseases (SD) and related conditions.

## Methods

A search strategy was conducted in Pubmed by using MESH terms, in LILACS and SCIELO using DECS terms to identify studies on PD prevention. The search strategy was as follow: (“Hispanic or Latino”[Mesh] OR “Caribbean Hispanic people” [Supplementary Concept]) AND “Periodontal Diseases”[Mesh]) AND “prevention and control” [Subheading]. A manual search strategy was also carried out specifying the name of each Latin American a Caribbean country. Systematic reviews (SR), cross-sectional and analytical studies and until December 2023 were included, without language restriction. Documents on health policies of each country and the region were reviewed. The opinions and recommendations of experts were considered for the recommendations and regional action plan. The articles were selected using title and abstract screening. All eligible studies were read in full text and were considered by all authors for citation within the final text.

### Evidence and recommendations on social determinants, risk factors and behavioral changes regarding PD over the life course of individuals lives

#### Evidence of public and private policies on periodontal prevention in Latin America and the Caribbean (community level)

The WHO Commission on Social Determinants of Health emphasized the importance of socioeconomic, political, and environmental factors of health: the circumstances in which people are born, grow, live, work, and age. The LATAM and Caribbean population is diverse in terms of sociodemographic determinants, economic and social inequality, and exposure to risk factors and systemic conditions. These circumstances determine the behaviors that people adopt and the possibilities of modifying habits. Few community strategies have integrated periodontal prevention into their daily public and private practice according to conditions of inequality, income, educational level, and access to health services.

**Figure 1:**
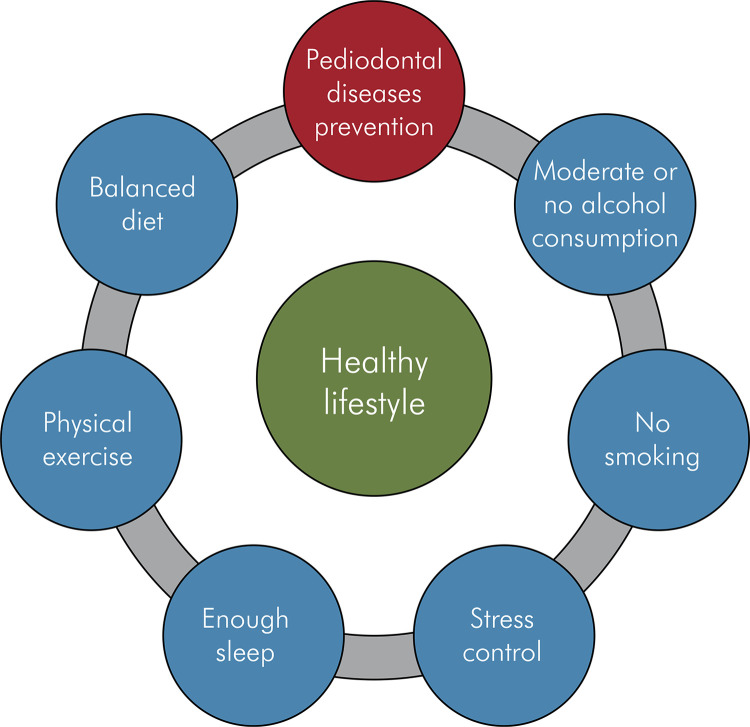
Healthy lifestyle goals, with the participation of periodontal health as a goal for the region.

Community preventive strategies for PD should not be isolated from health programs with the aim of establishing healthy lifestyles. Due to its status as a low-grade inflammatory disease, it should be considered, especially in more vulnerable populations. Therefore, community strategies public and private health systems need to provide community strategies that involve oral health in risk control programs for cardiovascular and metabolic diseases, pregnant women, etc. Furthermore the prevention of PD should be integrated into the strategies for promoting and communicating information on acquiring healthy lifestyles, such reducing tobacco consumption, adopting healthy nutrition, physical exercise, improvement in sleep, moderate alcohol consumption, and strategies to manage stress^5^ (Figure 1) Most experts believe that state public and private health policies should not only be maintained, but they should also be improved. Government and private entities, scientific associations and universities should act together to improve the periodontal health and well-being of the population. Strategic alliances with Dental Product Companies are necessary to achieve the Distribution of free or affordable oral hygiene products to underprivileged areas.

#### Role of scientific associations and universities in the primary prevention of periodontal diseases (community/individual level and recommendations for research)

Providing optimal periodontal health represents a challenge to the dental profession, scientific associations, and dental education institutions. The Iberoamerican Federation of Periodontology (FIPP) held a Latin American consensus, using Delphi Methodology, with experts from 16 countries to discuss trends in Periodontics for the year 2030.^6^ As regards public health policies and frequency of prevention and treatment procedures performed in periodontics in the public sector, no consensus was found. “Oral hygiene” achieved a moderate consensus. However, no consensus was reached regarding the use of interproximal brushes. Nevertheless, experts considered that the recommendation of other interdental cleaning devices will increase (71.6%). There was a consensus on the significant systemic connection with PDs and the need for collaboration between doctors and dentists. In contrast, there was no consensus relative to the role of public health in periodontal diseases, which may reflect differences in the public health systems of participating countries. Most experts believe that state public health policies should be maintained, but continue to see the need to increase not only prevention activities by the private sector, but also increase e/efforts to raise awareness about PDs.

**Figure 2:**
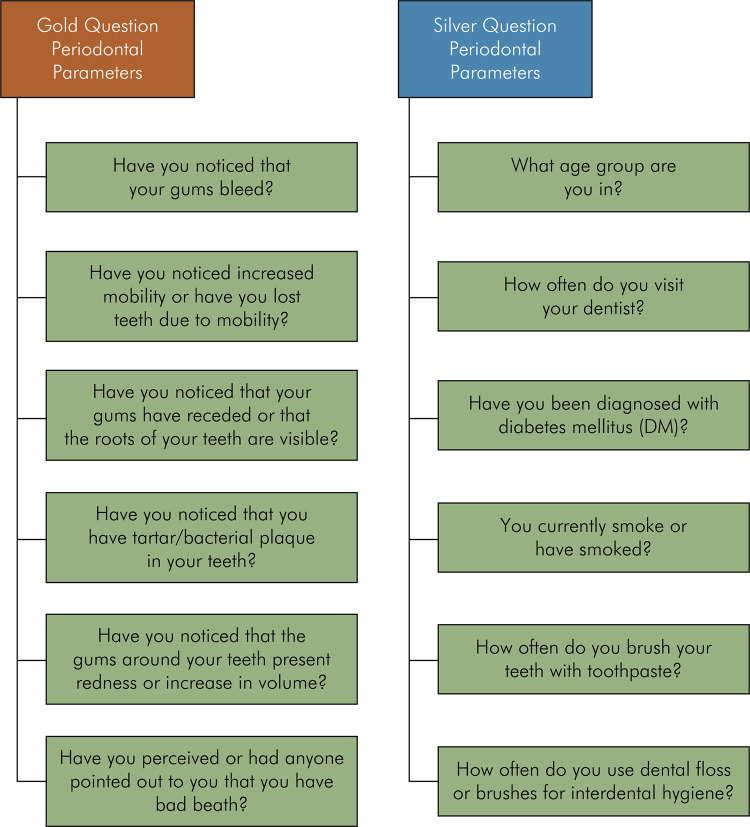
Gold and silver questions from the perio-awareness APP to facilitate patient/dentist empowerment.

A communication strategy on social networks among health professionals reported an increase in interest of over 90% in topics, such as: the importance of oral health as a healthy lifestyle, recognition of risk factors shared with chronic non-communicable diseases and importance of periodontal care to benefit systemic health.^7^ An Manifesto by LAOHA in conjunction with FIPP promoted the dissemination of content on social networks ,directed towards health professionals and patients, on early signs of PD, self-care, strategies for controlling CRF with other NCDs and the connection with SD.^8^ An online application initiative (perio-awareness) was published to promote awareness about the prevention of PD. The application evaluates 12 parameters (“6 gold and 6 silver Questions”) that patients can assess their periodontal signs and symptoms. Based on an algorithm, recommendations are given to encourage the search for a professional diagnosis and an appropriate patient/professional interaction. The recommendations provided by this application are based on the survey finding about the possible characteristic parameters/situations/habits of users^9^ (Figure 2).

#### Evidence on the preventive impact of controlling risk factors related to periodontal diseases

The WHO (World Health Organization) has recommended the development of joint preventive strategies for many NCDs due to shared common risk factors (CRFA). In the region, however, there is limited evidence as regards this type of approach and the use of personalized medicine.^10^ In PD, socio-environmental risk factors such as smoking, alcohol consumption, unbalanced diet, poor hygiene, inadequate access to dental services, stress, and occlusal trauma should be prioritized in health care.^11^ Successful outcomes in preventing and reducing tobacco consumption in LATAM and the Caribbean must be analyzed and replicated for the prevention of other conditions and diseases. In the region, educational strategies for pregnant women have been implemented, demonstrating an increase in knowledge, practices, and empowerment for the control of PD and other risk factors.^12^

The authors summarized a consensus recommendations/call to action on the impact of preventive measures on social determinants, risk factors and behavioral changes in relation to PD throughout the life-course of individuals (Table 1).

**Table 1:** Recommendations on social determinants, risk factors and behavioral changes as regards PD over the life -course of individuals.

Questions?	Recommendations on social determinants, risk factors and behavioral changes relative to PD over the life-course of individuals	Call to action level
What strategy is required to empower the medical profession about the importance of PD prevention?	Community level:	CLR
	It is necessary to increase efforts to raise awareness about PD so that health authorities pay more attention to the importance of periodontal health.	
	Develop and Implement (CPG) to advocate for periodontal health across all life stages, emphasizing risk management and adherence to lifestyle	
Who should lead implementation of these preventive strategies at the community and individual levels?	Community level:	CLR
	The participation of leaders in the region, who participate in public health policy decisions is required to link periodontal health into risk factor control programs and adherence to healthy lifestyles.	
	Individual level:	ILR
	The dental office must become an additional setting where patients can learn about healthy lifestyles.	
	Establish a collaboration network among dental and medical professionals to share knowledge, research findings, and educational resources on periodontal and systemic health interrelations.	
	Emphasize Personalized Oral Hygiene Education: Stress the importance of tailored oral hygiene instructions in professional development sessions for dental practitioners, catering to the diverse needs of patients including those with special needs.	
What is the research priority for the region on this topic?	Community level:	RR
	Invest in studies exploring the impact of mobile health applications, tele-dentistry, and social media on oral health awareness and self-care. Focus on assessing how these technologies improve access to dental care for marginalized communities.(Perio-awareness APP Assessment)	
	Execute comprehensive campaigns across community settings such as schools, workplaces, and public spaces, using both traditional and digital media to highlight the risks of PD and its impacts on systemic health. Engage local influencers to help with extending the reach and effectiveness of campaigns.	

CLR: community level recommendation; ILR: individual level recommendations; RR: recommendations for research.

## Evidence and recommendations on the impact of mechanical and chemical control of bacterial plaque and bleeding for the prevention of gingivitis

### Evidence on the effectiveness of mechanical and chemical preventive strategies (community level)

The primary preventive action at community level involves health education programs to raise awareness among individuals at different stages of life, about the necessity and importance of daily oral hygiene care. To achieve this, these programs need to be implemented in different settings, such as schools and both public and private healthcare services, to reach the widest audience possible. However, the challenge to preventive actions at population level is often related to access to healthcare services. Implementing effective educational programs to promote oral hygiene practices and providing easy access to dental services pose significant challenges. The lack of resources, adequate infrastructure, and awareness within communities can limit the effectiveness of preventive interventions.

### Evidence on the effectiveness of mechanical and chemical preventive strategies (individual level)

Daily habits play a crucial role in the effective control of supragingival biofilm. Although brushing is considered an essential method, there are still some questions related to the recommendation of specific characteristics of manual brushes, use of electric brushes, and the choice of interproximal devices. In a systematic review evaluating mechanical control in individuals undergoing periodontal maintenance, 80% of studies comparing manual and electric toothbrushes found no significant differences between them. However, the authors emphasize the lack of robust evidence due to the limited number of studies.^13^ As regards bristle design, toothbrushes with tapered tips showed superior reductions in the plaque index and gingival bleeding compared with round-tipped brushes.^14^ Relative to electric brushes, oscillating-rotating powered toothbrushes exhibited a slight difference in plaque control and improvement in gingival health when compared with frequency sonic powered toothbrushes.^14^ Moreover, more importantly than a technique, it should be emphasized that strategies must be taught and communicated to patients according to individual characteristics or their stratification according to risk.

A multicenter study on oral health behavior in South American adults showed that 84.2% of subjects brushed their teeth twice a day or more, but only 17.7% reported interproximal cleaning daily.^15^ Interproximal cleaning devices, especially dental floss, are more effective when combined with manual brushing than manual brushing alone. There is limited and inconsistent evidence for tooth cleaning sticks and oral irrigators,^16^ although interdental cleaners with rubber bristles were preferred by study participants.^17^ When prescribing mechanical control items, patient skills and preference should be considered, especially in older adults dealing with xerostomia and have low manual ability.

In SR, complementary antiseptics in mouthwashes and toothpaste have been reported to provide statistically significant reductions in gingival, bleeding and plaque indices, and combination of the two has shown better results.^18^ Recent studies have suggested reevaluation of oral health prevention and promotion. Recent research has focused on the effectiveness of fluoride toothpaste and other antimicrobial agents such as stannous fluoride-based toothpaste and sodium bicarbonate toothpaste. A SR found that stabilized SnF2 toothpaste had a positive effect on reducing the accumulation of dental calculus, dental plaque, gingivitis, stains and halitosis.^19-21^ This intervention should be explored as a preventive aid in the progression of plaque-induced gingivitis to periodontitis.

Essential oils and cetylpyridinium chloride (CPC) mouthwash were the active ingredients most frequently used in preventing gingivitis. Overall, studies have shown improvements in the clinical parameters evaluated.^18^ When compared with a placebo solution, CPC demonstrated good efficacy for plaque and gingival inflammatory parameters on interproximal surfaces.^22^ Recently, a multi-component oral care regimen with a zinc formulation (Dual) and CPC + In mouthwash was shown to be effective in reducing gingival inflammation and supragingival biofilm in patients with gingivitis.^23^

### Evidence on the effectiveness of preventive strategies according to the life-course of the population (community/individual level and recommendation for research)

In the region, there is an unclear personalized or stratified prevention strategy throughout individuals’ lives, with limited participation from other health professionals. In children and adolescents, prevention has been focused on dental caries, and periodontal health is not always considered. In the early life stages, children’s limited autonomy emphasizes the active role of parents and caregivers in promoting healthy oral hygiene habits. Studies exploring mechanical methods for school-age children have demonstrated positive outcomes with manual, differently shaped, or electric toothbrushes.^24,25^ Customizing toothbrush handles enhances hygiene standards for Down syndrome children.^26^ Alternative motivational methods, including musical videos or verbal instructions, have also demonstrated improvements in plaque and gingival indices.^27,28^ The literature remains uncertain about chemical control for preventing gingivitis in this age group.

In adolescence, many individuals in the region undergo orthodontic treatment without supervision for periodontal health, which is a decisive moment for future periodontal health and bone support. A SR revealed that orthodontic manual brushes outperformed conventional manual brushes in plaque removal, with no significant difference in gingival bleeding.^29^ Electric toothbrushes, compared with manual types, demonstrated a significant reduction in both plaque and gingival indexes.^30^ SR comparing different mouthwashes for patients with fixed orthodontic appliances revealed similar results, indicating that chlorhexidine was effective in reducing biofilm and signs of gingival inflammation.^31,32^

In young adults, only programs for pregnant women have prioritized the diagnosis and prevention of periodontal diseases. One study reported that the consumption of L. reuteri lozenges may be a useful complement in the control of pregnancy gingivitis.^33^ In some countries, they take PD in diabetes into account in their clinical practice guidelines for diabetic patients, but they are clear about both the diagnostic procedures (including self-diagnosis) and the clinical management of the disease. It has been suggested that probiotics may provide additional benefits to the periodontal and peri-implant parameters in patients with type 2 diabetes.^34^ A SR suggested that probiotic supplementation improves clinical parameters, reduces pathobionts and proinflammatory markers in patients with PD. However, there is a lack of evidence on its role in primary prevention and its impact at the community level. Another SR showed a slight improvement in the inflammatory clinical parameters of patients treated with the use of probiotics in an experimental gingivitis model.^35^ As the population survival rate increases, more people will need preventive and even peri-implant periodontal care. The above-mentioned situation adds to a greater probability of NCDs in adults and older adults. In the elderly, prosthetics and medical conditions significantly impact prevention strategies. A study characterized the oral health of elderly individuals as precarious, with nearly half requiring assistance for hygiene care. Additionally, challenging access to dental care underscores the importance of preventive measures.^36^ Investing in health education for family members, caregivers, and nursing staff, along with the presence of a dental surgeon in elderly care institutions or hospitals, and organization of systematic care are deemed crucial.^37-39^ The authors summarized a consensus recommendations/call to action on the impact of mechanical and chemical control of plaque and bleeding for the prevention of gingivitis (Table 2).

**Table 2:** Recommendations on the impact of mechanical and chemical control of bacterial plaque and bleeding for the prevention of gingivitis.

Questions	Evidence and recommendations on the impact of mechanical and chemical control of bacterial plaque and bleeding for the prevention of gingivitis	Call to action level
What is the role of university education and scientific associations?	It is essential to implement educational programs and modern communication strategies (APP, social networks) in schools and public/private health services to disseminate information about the importance of mechanical and chemical control of dental biofilm, prebiotics and probiotics at different stages of life and its impact on the quality of life of children during their growth and development. This approach will facilitate community campaigns with the aim of promoting healthy oral hygiene habits.	CLR
	Integrate oral health education into schools and community initiatives, offering practical demonstrations of effective oral hygiene practices. Organize health fairs providing free dental screenings and educational workshops to emphasize the significance of oral hygiene.	
What is the role of private oral product companies in PD	Ensure access to a variety of toothbrushes, interproximal devices, toothpaste, and mouthwashes in all communities, particularly in the more vulnerable populations, by means of public health policies that involve the state joining with oral products companies. Furthermore, specialized products that demonstrate benefits should be available at low costs in patients with a history of more advanced stages advanced stages of gingivitis and periodontitis.	CLR
	Transform dental office waiting areas into educational hubs with resources such as videos, brochures, and interactive tools promoting mechanical and other adjunctive preventive strategies, such as prebiotics, probiotics and antimicrobial properties of the mouthwashes and dentifrices.	
What is the priority in research on this topic for the region?	It is essential to conduct research that assesses the efficacy of different prevention methods, across various age groups. Moreover, these studies should incorporate more patient-measured outcomes, focusing on the individual’s perception of the products.	RR
	It is necessary to evaluate the effectiveness of different formulations and concentrations of antimicrobial agents in dentifrices and mouthwashes; doses and use of probiotics and prebiotics.	

CLR: community level recommendation; ILR: individual level recommendations; RR: recommendations for research.

### Evidence on the impact of PD preventive strategies for the management of systemic diseases and conditions that are related to periodontal diseases

There is strong evidence showing that people with periodontitis have an increased risk of dysglycemia, insulin resistance and higher HbA1C levels in patients with periodontitis and diabetes mellitus.^40^ In the Colombian and Brazilian populations, periodontitis was associated with metabolic syndrome (MetS) and glucose intolerance.^41,42^ In a multicenter cohort study in Brazil, an association of PD with subclinical atherosclerosis was established.^43^ Likewise, a relationship between periodontitis, severe periodontitis and Acute Coronary Syndrome was shown in Colombian and Chilean individuals .

Pregnant women with periodontal disease have shown an increased risk of adverse pregnancy outcomes (APOs), including preterm birth (PTB), low birth weight newborns, preeclampsia, and gestational diabetes mellitus (GDM). In Colombia, a study reported an association between the severity of periodontitis and pre-eclampsia (OR: 3, 95%CI: 1.91–4.87; p < 0.001).^44^ Another study suggested an association between periodontal pockets and APOs in low-income pregnant women, highlighting factors such as threatened abortion, absence of antenatal care, hypertension, chronic infections, and periodontal diagnosis as important conditions associated with APOs.^45^ Similarly, in Chile, a study with 870 pregnant women reported an association between gingivitis and PTB, with significant reductions in PTB observed in pregnant women who received periodontal treatment. Likewise, a recent study screening 1,086 pregnant women in Chile showed a high prevalence of periodontitis (73.1%), with a low percentage of patients with healthy periodontium or gingivitis (26.9%).^46^ Other studies also suggested that pregnant women with gestational diabetes mellitus exhibited a worse periodontal condition than those with healthy pregnancies, measured by periodontal probing depth, clinical attachment loss, and bleeding on probing. Furthermore, the inflamed periodontal surface area was larger in those with GDM pregnancies.^47^ Moreover, obesity was related to periodontitis severity, with a relative risk ratio (RRR) of 1.66 (95%CI: 1.05–2.64; p = 0.03) and 1.57 (95%CI: 1.09–2.27; p = 0.015) for stage III periodontitis compared with those with periodontal healthy/gingivitis and stage II periodontitis, respectively.^46^ However, these findings are not consistent across all Latin American countries. For instance, a Brazilian study was unable to demonstrate the association between the severity of periodontal disease and APOs.^48^ The differences between the studies could be explained due to ethnic and socioeconomic variations, the prevalence and severity of periodontitis, case definitions, population heterogeneity, different health systems and monitoring the oral health care of pregnant women.

### Evidence on the effectiveness of preventive strategies based on the association of periodontal diseases with systemic conditions (community level)

In recent years, a positive aspect has been the implementation of public policies, including clinical practice guidelines and comprehensive care routes directed towards pregnant women in LA. There are many beliefs about the safety of dental procedures during pregnancy, a lack of knowledge of the impacts on adverse pregnancy outcomes and oral health, and the need for oral hygiene during pregnancy, factors that discourage access to health services.^49^ However, in LA, the public health ministries of Argentina, Brazil, Colombia, Chile, Ecuador, Peru, and Uruguay, among others, have established primary care programs for pregnant women in their countries, however, there is still no evidence of the impact of these programs on the community. Despite the extraordinary efforts of governments to implement these priority primary care programs, access to health programs continues to be limited.

In some countries, chronic patients are a priority in primary prevention, but it was not possible to completely identify the role and detailed care guidelines for health personnel relative to the control of periodontal disease. Many people from different populations, who have chronic diseases ,and have a high prevalence of periodontitis, do not, however, go for dental visits regularly. Medicare service showed a lack of preventive dental care in African Americans/Black and Latin Americans, with lower educational attainment and household income, who suffer from diabetes, prediabetes, hypertension, and are smokers.^50^

Several studies were conducted to establish the impact of regular oral hygiene on the prevention of chronic diseases. In a 10-year cohort study of the National Health Insurance System-Korea Health Screening Cohort, tooth brushing ≥ 3 times/day showed a protective effect against new-onset of diabetes and on reducing blood sugar levels. HbA1C. The use of antiseptic products for plaque control in patients with NCDs and other medical or hospital conditions is controversial. Oral care in hospitalized patients in critical and non-critical conditions is recommended in the CPG to maintain oral health. Some systematic reviews, including Cochrane reviews, have shown that chlorhexidine reduces the risk of ventilator-associated pneumonia (VAP).^51,52^There is controversy about the role of chlorhexidine in the incidence of mortality, and the evidence is weak.^51,53,54^

The authors summarized a consensus recommendations/call to action on the impact of preventive strategies for the management of SD and PD related conditions (Table 3).

**Table 3:** Recommendations on the impact of preventive strategies for the management of systemic diseases and periodontal disease-related conditions.

Questions	Evidence and recommendations on the impact of preventive strategies for the management of systemic diseases and periodontal disease-related conditions.	Call to action level
What is the importance of communication strategies for the multidisciplinary management of systemic diseases and PD?	Community level:	CLR
	Educational programs directed towards the community on the benefits of attending oral health services during pregnancy.	
	In addition, those women who wish to become pregnant need to be identified so that they can anticipate a pregnancy without periodontal disease.	
	Patients with other conditions such as cardiovascular and metabolic disorders need to have more emphasis is on the prevention of PD.	
What communication strategies should be implemented by periodontists in the region?	Individual level	ILR
	It is necessary to form a regional group of experts to contact regional and local scientific societies in medicine such as the regional network of the International Diabetes Federation, other medical associations, and health faculties.	
	Build Partnerships with Patient Associations: Work with patient organizations to spread information about periodontal disease prevention and its connection with systemic health, customizing educational materials for diverse community needs.	
	Train Dental Professionals in Patient Communication: Equip dental professionals with the skills to effectively communicate the connection between oral health and overall wellness and tools promoting healthy lifestyle choices.	
What is the priority in research on this topic for the region?	Create care models that integrate oral health assessments into routine check-ups for individuals with chronic diseases or during pregnancy. Offer training on the systemic implications of periodontal health to non-dental healthcare providers.	RR
	It is necessary to promote multicenter research that assesses the role of PD prevention with different approaches (mechanical therapy, antiseptics, foods with prebiotics functions, and probiotics tablets) and its role in the onset or control of systemic conditions.	

CLR: community level recommendation; ILR: individual level recommendations; RR: recommendations for research.

## Research gaps, conclusions, and future needs

Evidence on the prevention of PD, qualitative research and behavioral changes continues to be scarce, at both the individual and community levels. In preventing periodontal diseases, the human factor plays an important role, that is, the physical, psychological, and social characteristics that affect human interaction with other people, in this case, the healthy or periodontally compromised patients and health professionals. Another challenge in the region is that each individual or community has unique social determinants and/or environmental and biological aspects that must be identified to enable the success of preventive strategies.^55^ The term situational awareness requires an understanding of the environment, and ability to anticipate variations in the health/disease process, which is why it is necessary to educate more empowered professionals, who are trained in behavioral sciences.

This consensus is a call for action to network between scientific societies, Universities, LAOHA, FDI, WHO/PAHO and the International Association for Dental Research (IADR), public and private entities, in order to develop research projects, unify and disseminate strategies, preventive measures, and CPG in periodontics. and to evaluate their clinical impact on oral and systemic health in different populations.

In the future, with the increasing availability of large datasets that integrate cellular and microbial genomic information, information on organ function, aspects of the oral cavity and saliva, and behavioral patterns in populations, it will be possible to stratify population groups for the purpose of estimating individual risks for their periodontal health and empowerment needs more accurately.
